# Composition and structure of magnetic high-temperature-phase, stable Fe–Au core–shell nanoparticles with zero-valent bcc Fe core†

**DOI:** 10.1039/d0na00514b

**Published:** 2020-08-10

**Authors:** Marius Kamp, Anna Tymoczko, Radian Popescu, Ulrich Schürmann, Ruksan Nadarajah, Bilal Gökce, Christoph Rehbock, Dagmar Gerthsen, Stephan Barcikowski, Lorenz Kienle

**Affiliations:** Institute for Materials Science, Synthesis and Real Structure, Kiel University Kaiserstraße 2 24143 Kiel Germany lk@tf.uni-kiel.de; Technical Chemistry I, Center for Nanointegration Duisburg-Essen (CENIDE), University of Duisburg-Essen Universitätsstrasse 7 45141 Essen Germany; Laboratory for Electron Microscopy (LEM), Karlsruhe Institute of Technology (KIT) Engesserstr. 7 76131 Karlsruhe Germany

## Abstract

Advanced quantitative TEM/EDXS methods were used to characterize different ultrastructures of magnetic Fe–Au core–shell nanoparticles formed by laser ablation in liquids. The findings demonstrate the presence of Au-rich alloy shells with varying composition in all structures and elemental bcc Fe cores. The identified structures are metastable phases interpreted by analogy to the bulk phase diagram. Based on this, we propose a formation mechanism of these complex ultrastructures. To show the magnetic response of these magnetic core nanoparticles protected by a noble metal shell, we demonstrate the formation of nanostrands in the presence of an external magnetic field. We find that it is possible to control the lengths of these strands by the iron content within the alloy nanoparticles.

## Abstract 2

Advanced quantitative TEM/EDXS methods were used to characterize different ultrastructures of magnetic Fe–Au core–shell nanoparticles formed by laser ablation in liquids.

## Graphical abstract

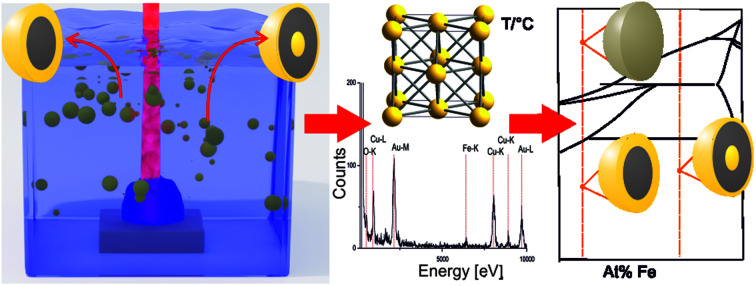

## Introduction

**Fig. 1:**
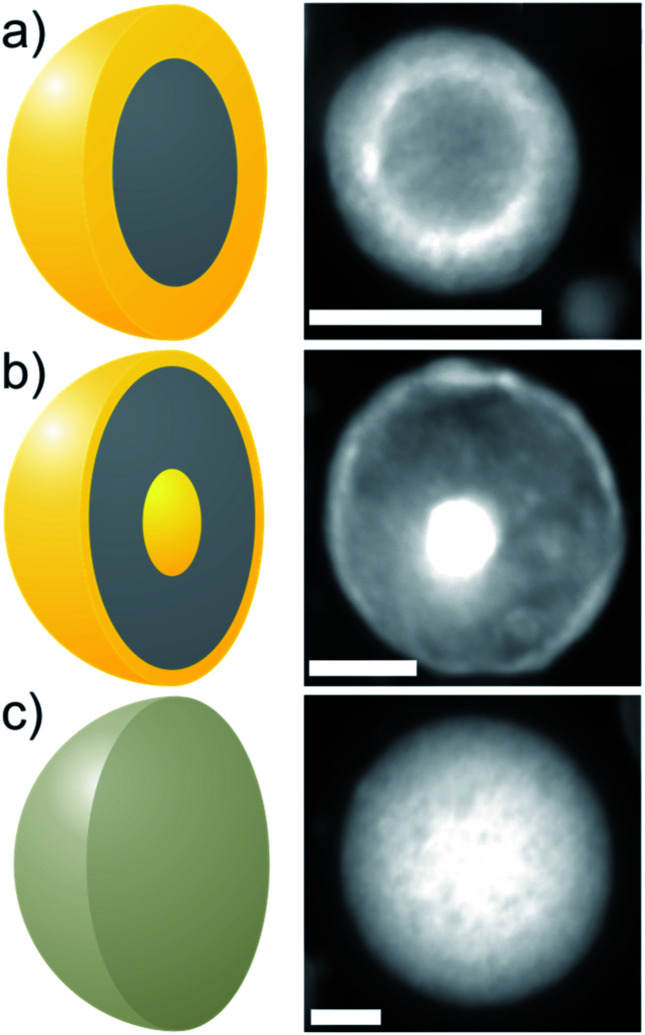
Classification of the three synthesized ultrastructures of Au–Fe NPs: HAADF-STEM *Z*-contrast images of CS (a), NCS (b), and SoSo (c) NPs and associated sketches (each left); scale bars are 25 nm.

Bimetallic Au–Fe nanoparticles (NPs) with complex internal phase structure (ultrastructure), like core–shell (CS) (an elemental Fe core surrounded by an Au shell (Fig. 1a)), and nested core–shell (NCS) NPs (an Au core embedded in Fe surrounded by a thin Au shell (Fig. 1b)), combine the properties of their constituents. The ultrastructure of the NPs and the chemical composition of their components influence the performance in diverse application fields.^1–5^ Consequently, these NPs may be used, *e.g.*, in terms of magneto-plasmonic properties, electrocatalytic oxygen evolution,^6^ or MRI/optical dual imaging, for medical applications.^7^ The use of magnetic alloy NPs as electrically conducting entities within a composite material presents a further promising application area in fields such as microelectronics, medicine,^8^ or even transparent conductive coatings.^10^ In comparison to typically used carbon nanotubes or indium tin oxide, magnetic NPs can be aligned by an external magnetic field during polymer processing^9^ to form potentially conductive strands within the polymer, while maintaining high transparency.

In contrast to wet chemical methods, laser ablation in liquid (LAL) allows the scalable^11^ synthesis of complex metastable NPs^3,12,13^ without the presence of chemical ligands or residues of reducing agents on the surface. In the case of the Au–Fe system, solid solution (SoSo) and phase-segregated NPs were observed with a multitude of ultrastructures like CS with a Fe core protected from oxidation by an Au-rich shell.^14^ Also, unique ultrastructures formed in the process, such as NCS NPs with an ultrathin, crystalline Au shell,^15^ which have not been fully characterized to date.

The formation of segregated NPs in LAL is based on the minimization of interface and surface energy by a thermodynamic driving force, which was also verified by Amram *et al.*^16,17^ On the other hand, non-equilibrium steps such as the initial steps of LAL with unparalleled cooling rates could yield metastable particle structures not thermodynamically favored at room temperature.^3,18^

Recently, we identified that a Fe-rich target composition and a NP diameter above 10 nm are decisive for the formation of CS NPs.^12^ Furthermore, the CS yield could be increased up to 99% in mass, by utilization of nanosecond pulses and thin layered targets.^13^ The internal phase structure of these materials has not been fully elucidated yet, *e.g.*, contradicting data on the crystal structure (bcc *vs.* fcc), and the chemical composition of the Fe-rich core exists.^12^*In situ* heating experiments exhibited fundamentally different thermodynamically stable morphologies based on the chemical composition.^19^ Besides, for specific chemical compositions, a complex segregated ultrastructure (NCS) was discovered, which has not been characterized yet. Mechanistic understanding necessitates determining the composition and crystal structure of core and shell on the single-particle level. Furthermore, doping of a magnetic Fe core with non-magnetic atoms such as Au as well as introducing a non-plasmonic metal like Fe to a plasmonic Au shell could seriously hamper the potential magneto-plasmonic properties of these particles, which necessitates a thorough study of their ultrastructure.

The application of indirect methods, such as electron diffraction, X-ray diffraction, and magnetic characterization, only yield mass-weighted information of the sample but do not allow the quantification of the chemical composition at the nanoscale on a single particle level. However, such information is vital to understand *e.g.* the protective nature of the Au shell and other properties like plasmon resonance, which can be affected by the Fe content. Furthermore, the in-depth characterization of the chemical composition of individual NPs, including their components, *e.g.*, shell and core, would lead to valuable insights and could substantiate formation models for the multitude of ultrastructures. An in-depth characterization of these nanostructures, *e.g.* separation between CS and NCS NPs is only possible using transmission electron microscopy (TEM) in combination with advanced sample preparation. One suitable method for the analysis of multi-component systems for TEM is based on the preparation of cross-sections using a focused ion beam (FIB), thus minimizing the superposition of, *e.g.*, the shell and core-forming components. An advancement in TEM preparation by Vieweg *et al.*^20^ allows site-specific sectioning *via* scanning electron microscopy (SEM) and FIB.^21,22^ However, in the present case, the selection of nanostructures based on their ultrastructure, *e.g.* CS and NCS NPs, is only possible using TEM. Therefore, a unique TEM based method for the determination of the composition of single components at the nanoscale could be the basis for a fundamental understanding of segregation processes in ultrastructures.

In this work, we applied EDXS in combination with high-angle annular dark-field (HAADF) scanning transmission electron microscopy (STEM) and an advanced data evaluation technique, which allows the quantification of the chemical composition of single NPs. In this approach, EDXS line profiles yield chemical compositions of single NPs on a 1 nm scale, which are processed by a subshell model to eliminate the superposition of core and shell contributions, resulting in the chemical composition of the individual components of segregated NPs. This study describes the application of the advanced subshell evaluation technique to characterize different metastable ultrastructures formed by laser ablation in liquids. These studies allow to elucidate elemental compositions on the nanoscale and help to clarify the formation mechanism of these metastable NPs.

## Experimental

The NPs are produced by the one-step synthesis of laser ablation in liquid. A pulsed laser beam ablates an alloy target in a solvent and synthesizes NPs. Two lasers are used for the synthesis: an 8 ns Nd:YAG laser (Rofin-Sinar) at 1064 nm with a repetition rate of 15 kHz and a nominal fluence of 3.85 mJ cm^−2^ as well as a 10 ps Nd:YAG laser (Ekspla) at 1064 nm with a repetition rate of 100 kHz and a nominal fluence of 3.1 mJ cm^−2^. For both systems, a lens with a focal length of 100 nm was used to focus the beam through a glass window into a batch chamber containing the Au–Fe alloy target. The samples are generated in 30 ml 99.8% acetone (Carl Roth GmbH, Karlsruhe) or 30 ml 3-pentanone. The NPs are examined in high-angle annular dark-field (HAADF) scanning transmission electron microscopy (STEM) mode, which enables atom number-dependent *Z*-contrast next to mass thickness contrast. Au_50_Fe_50_ produced in acetone with the picosecond laser and Au_20_Fe_80_ produced in 3-pentanone and acetone with the nanosecond laser. All TEM samples are prepared at room temperature in the air by drop-casting of a diluted nanoparticle (NP) colloid onto an ultrathin amorphous carbon film (3 nm) on holey carbon support film mounted on 200 μm mesh Cu grid (Plano GmbH). The TEM investigations are performed on a Tecnai F30 STwin G^2^ with a 300 kV accelerating voltage. Energy-dispersive X-ray spectroscopy (EDXS) measurements are performed with a Si/Li detector (EDAX system).

An FEI Osiris ChemiSTEM microscope operated at 200 keV electron energy was used for HAADF-STEM imaging. For performing EDXS experiments, the instrument is equipped with a Bruker Quantax system (XFlash detector). EDX spectra are quantified with the FEI software package “TEM imaging and analysis” (TIA) version 4.7 SP3. Using TIA, element concentrations were calculated based on a refined Kramers' law model, which includes corrections for detector absorption and background subtraction. Sample preparation by focused-ion-beam milling system was performed with a FEI Helios NanoLab system using the lift-out method. Prior to FIB preparation, the NPs were embedded in a carbon matrix by a carbon injector.

To produce Au–Fe nanostrand-polymer composites, the approach described in Barcikowski *et al.*^9^ was followed. First, a solution of 5 wt% poly(methylmethacrylate) (PMMA) with Au–Fe NPs in acetone was prepared. A NP concentration of 0.2 wt% in acetone was used. The Au–Fe nanoparticle-containing poly(methylmethacrylate)-acetone solution was dried on a glass substrate under an external magnetic field, with a flux density of 150 mT. During drying, the formed nanostrands were measured using an optical microscope (CX 40, Olympus).

## Results

In this study, we will focus on the characterization of laser-generated CS (Fig. 1a) and NCS (Fig. 1b) Au–Fe NPs that are generated in addition to SoSo NPs (Fig. 1c) by LAL. We determined the morphology and the chemical composition of single NPs by HAADF-STEM combined with EDXS for two samples with a composition of Au_50_Fe_50_ and Au_20_Fe_80_. The EDX spectra obtained by scanning rectangular areas, including single NPs, are quantified to determine their (experiment-based) average chemical composition. Analyses of several single NPs show an average chemical composition that corresponds to that of the target (Table S1†). In Au_50_Fe_50,_ the variance (38.5) concerning the target composition is higher compared to Au_20_Fe_80_ (6), but no systematic deviation in terms of ultrastructure could be detected. These findings are supported by previous STEM-EDXS results,^19^ which indicate that the NPs on average have the chemical composition of the target. In particular, the measurement included several assemblies of a large number of NPs, which showed the target composition. The ultrastructure of the individual particles is thus not predetermined by their chemical composition. The overall composition of all examined particles is ruled only by the composition of the alloy target used during ablation. This seems to indicate that all structures found here emerge from a pre-stage, with full atomic mixing and overall compositions only determined by the laser ablation target.

**Fig. 2:**
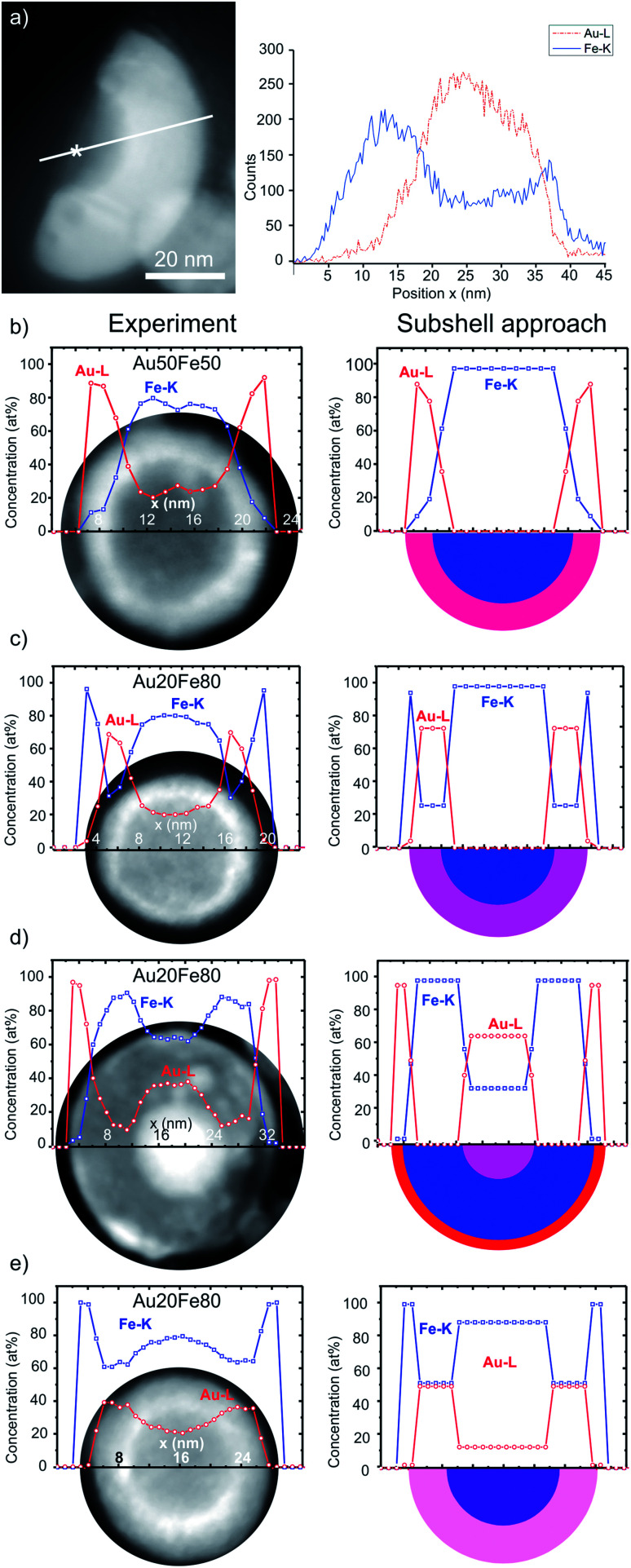
(a) HAADF-STEM *Z*-contrast image of a wedged cutout from CS NP with EDXS line scan direction indicated by a white line. An asterisk marks an exemplary core position with a measured chemical composition of 100 at% Fe (Fig. S3†). (b–e) On the left: elemental EDXS line scan (raw data) and HAADF-STEM *Z*-contrast images of the NP. On the right: line scan after processing by the subshell approach, yielding chemical compositions of individual components and sketches of NPs (half-sphere). The error is estimated to be of ±2 at%. The color code (blue: pure Fe, red: pure Au) is used to represent the chemical composition of different NP ultrastructures. The *x*-axis of the line scan matches the scale bars of HAADF-STEM *Z*-contrast images. Average chemical composition: (a and b) Au_50_Fe_50_ ns laser pulse duration, and (c)–(e) Au_20_Fe_80_ ps laser pulse duration.

Since the NPs in one sample have the same average chemical composition, a characterization of the individual components is necessary to determine the elemental distribution in various ultrastructures. In particular, analyses are necessary to study whether core and shell are composed of pure elements or alloys with variable chemical composition.^12^ One possible method is the preparation of cross-sectional TEM samples *via* a site-specific focused-ion-beam lift-out method.^20^ However, for the investigation of NP ultrastructure, the experimental procedure remains very challenging, because one needs to cut out a thin slice of a NP, to get rid of the superposition effects from core and shell (Fig. S1†). As a result, such sample preparation allows the quantification of the core composition and structure determination without the superposition of the shell. The experimental results (Fig. 2a) indicate the formation of a pure Fe core. In principle, this finding applies only to a single CS NP without statistical significance, but as a reference experiment with high reliability, it will be helpful for the validation of other methods described below.

Furthermore, an alternative and less time-consuming method is applied to study the chemical composition of NPs with complex structures. In this procedure, EDX spectra are recorded along a line scan through the center of the corresponding NP, resulting in concentration profiles of different chemical elements within a single NP. However, regarding the quantification of such EDX spectra, one has to keep in mind that the obtained compositions are averaged along the electron-beam direction, *i.e.*, the whole volume along the electron trajectory contributes to the detected X-ray signal by superposition. As a consequence, it is not straightforward to quantify the chemical composition of the individual components of complex ultrastructures, like the core of CS NPs, from such EDXS elemental line scans.

As a solution, a subshell division procedure was applied in this work for the determination of the composition of different regions within complex NPs, which is described in detail elsewhere.^23,24^ Preconditions for this method are an EDXS detector with high signal-to-noise ratio, high quantum efficiency, and a small probe size (<0.5 nm) of the electron beam. Moreover, the NP shape is approximated to be spherical without distinctive surface roughness.

The broadening of the electron beam due to the elastic and inelastic interaction with the NPs could be another source for the imprecision of EDXS analysis at the nanoscale. The Goldstein equation typically estimates beam broadening (*cf.* ESI†). The results indicate a beam broadening below 1 nm, *i.e.*, of 0.80 nm and 0.78 nm, after passing through NPs with the maximum diameter of 29 (Au_20_Fe_80_) and 23 nm (Au_50_Fe_50_), respectively. Considering the beam broadening, the step size of EDXS line profiles was adjusted to 1 nm to avoid overlap between EDXS signals from two adjacent regions. Furthermore, from the nature of this method, the number of subshells is not set to a fixed value, because the diameter of the NPs affects the number of subshells. A subshell thickness of 1 nm is chosen, thus, the number of subshells is given by the diameter of the NPs.

The quantification of the composition is based on the assumption that the particle shape is approximately spherical. To verify that this assumption is correct, we use the following procedure. The average composition of a single NP (experiment-based average) was compared to the calculated average composition of the same NP (model-based average) by adding up the composition of all subshells. An average deviation of 2.3 at% was obtained between the experimental and the model-based average composition, which shows that the particles are indeed (quasi-)spherical (*cf.* Table S2†).

The left column of Fig. 2b–e shows the Au (Au-L_α1_ line) and Fe (Fe-K_α1_ line) concentration profiles obtained by the quantification of the EDXS line scans before the application of the subshell approach overlapped with the HAADF-STEM *Z*-contrast images of the analyzed NPs. The right column provides the model-based quantification using the subshell approach^23^ and, additionally, a schematic cross-section (half-sphere) of the NPs. Accordingly, the right panel of Fig. 2b shows an Au_50_Fe_50_ CS NP and the corresponding chemical concentration profiles of Au and Fe. A remarkable result is the indication of a purely elemental Fe core formation. We note that CS represents the only type of ultrastructure found for this sample.

Furthermore, chemical compositions of Au–Fe alloy shells were evaluated, which vary in the dependence of their ultrastructure and as a function of the overall chemical composition. The Fe content in the alloy shell of the NP shown in Fig. 2b with the overall (target) composition Au_50_Fe_50_ is 15 at%, while it reaches 26 at% for the CS NP in Fig. 2c, with the overall composition of Au_20_Fe_80_. Such differences may significantly influence their magneto-plasmonic and catalytic properties.^6,25^

NPs with other variants of ultrastructures, which are shown in Fig. 2d and e, are only observed for the more Fe-rich Au_20_Fe_80_. These include NCS NPs (Fig. 2d) that form two Au-rich components with different Fe contents, *i.e.* ultra-thin shells with ∼3 at% Fe and additional nested Au alloy cores with ∼34 at% Fe. Besides, CS NPs with alloyed cores (∼90 at% Fe) and thick shells (∼50 at% Fe) are found (Fig. 2e).

**Fig. 3:**
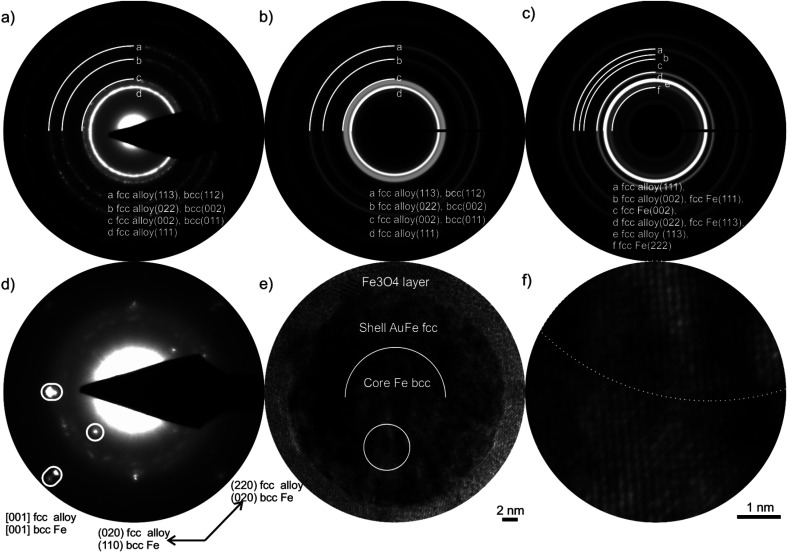
(a) Experimental SAED pattern taken from a NP agglomerate for Au_50_Fe_50_, which fits the simulated ED pattern of (b) a CS ultrastructure with Au-rich alloy shell and Fe bcc core with lattice parameters of *a* = 3.98 Å and *a* = 2.86 Å, respectively. The simulated pattern (c) for an fcc core with an alloy shell does not fit to the experimental results. (d) ED pattern of a single NP for Au_20_Fe_80_ with reflection splitting can be assigned to fcc alloy *a* = 3.9 Å, bcc Fe *a* = 2.90 Å, and Fe_3_O_4_ phases. (e) HRTEM micrograph of the same NP, circle, shows the position of (f) magnified interface of core and shell indicated by a broken line.

CS NPs depicted in Fig. 2c and e are covered by an outermost 3-rd shell containing only Fe and no Au. Based on further investigations, it can be attributed to a thin Fe oxide layer, which may be formed by an oxidation process in ambient air. The analysis of high-resolution TEM (HRTEM) micrographs and EDXS elemental maps (Fig. 3e and S2†) indeed reveals the formation of Fe_3_O_4_. Such oxide shells could also be generated by the accumulation of FeO_*x*_-containing residues when the particles are dried on the TEM grid. These residues could be further condensed on the surface of NPs through interaction with the electron beam. In the schemes in Fig. 2c and e, the 3-rd outer Fe oxide shell is not represented and will not take into account in the following, since it is probably a post-synthesis by-product that does not provide any insight into the NPs formation mechanism.

Nevertheless, it may be an essential finding when analyzing surface plasmon resonance or catalytic properties of NPs. The analysis of 23 NPs underlines that the oxidation only occurs for NPs with high Fe content in the shell. Accordingly, no NCS NP with an oxide shell was found, as the Au-rich shells of these NPs show a low Fe content. These results support the earlier reported process of chemical dealloying of NCS NPs requiring the formation of pinholes.^15^ Overall, the yet missing analytical proof is given by using an advanced electron microscopy method that the laser-generated Au_50_Fe_50_ CS NPs consist of an elemental Fe core and an Au–Fe alloy shell, with the shell probably having a concentration gradient that Au enriched on its shell surface.

Selected area electron diffraction (SAED) is used for crystal structure determination. However, SAED patterns contain superimposed data for complex NPs, including reflections of the components and double diffraction. Thus, only the combination of SAED, high-resolution TEM, and EDXS results can provide a full structural characterization, including the crystal structure, the composition of the individual NP components, and its crystallographic orientation relation (Fig. 3). Essential information for the determination of the crystal structure is the finding from the EDXS investigations that the core of Au_50_Fe_50_ CS NPs (Fig. 2b) does not contain a significant amount of Au. Only with this criterion, the crystal structure can be determined *via* its lattice parameters, since the lattice plane distances of alloyed and pure phases are only slightly different.

The validation of the fcc alloy shell and Fe bcc core for the Au_50_Fe_50_ target is given in Fig. 3a, which shows that the experimental results fit the simulated bcc-type core (Fig. 3b) but not to an fcc Fe structure. For an fcc Fe core structure, one reflection (002) with high intensity is absent in the SAED pattern (Fig. 3c). Also, we show that the core and the shell are single crystals by ED patterns and HRTEM micrographs of a single aligned NP (Fig. 3d–f). A HAADF-STEM *Z*-contrast image of the same NP verifies the CS ultrastructure (Fig. S4†). These detailed investigations have shown a particular orientation relation of the bcc core and the fcc alloy shell called Bain orientation,^26^ illustrated in Fig. 3d by split reflections. The structures of core and shell are rotated by 45° around [001], resulting in a Bain orientation relationship {001} fcc ∥ {001} bcc and <011> fcc ∥ <001> bcc. However, the ultra-thin shell of an NCS NP is polycrystalline, and no orientation relationship with the inner Fe shell or Au-rich core exists (Fig. S5†). These findings seem to contradict previous results by XRD analysis of Au_50_Fe_50_ NPs, where no XRD reflections from bcc Fe could be detected, solely Au-rich FCC structures.^12^ This is probably attributed to the fact that XRD is a mass-dominated characterization technique, and more mass abundant fcc structures could have overshadowed bcc reflexes in the analyzed samples.

**Fig. 4:**
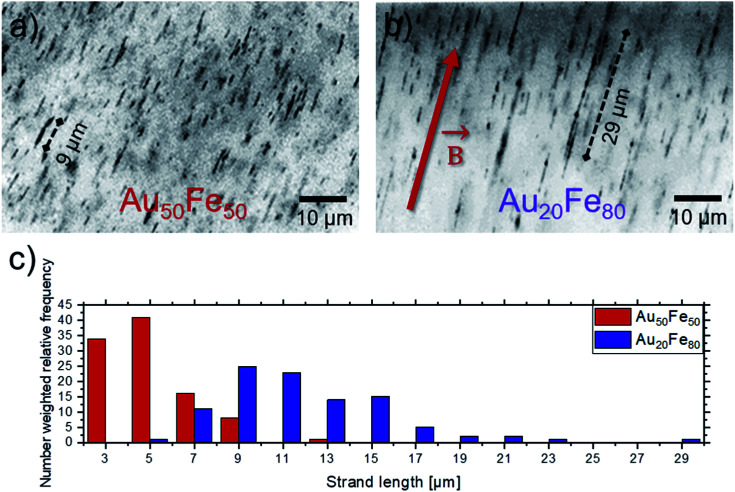
Magnetic-field induced Au–Fe-nanostrand formation in a polymer. Light optical microscopy images of the composite containing (a) Au_50_Fe_50_ and (b) Au_20_Fe_80_ nanostrands. The red arrow denotes the direction of the magnetic field. Also marked are some of the longest strands for each composition; (c) quantification of the nanostrand length as extracted from microscopy images for both compositions by image analysis.

To show the magnetic response of the studied Au–Fe alloy NPs, Au–Fe nanostrands are formed by the application of an external magnetic field (150 mT) during the formation of PMMA-Au–Fe composites (Fig. 4). Due to this magnetic field, the magnetized NPs exhibit a local magnetic field, which results in the attraction of the particle and alignment into strands. Both studied compositions, *i.e.*, Au_50_Fe_50_ and Au_20_Fe_80_, are forming strands, with maximum strand lengths of 13 μm and 29 μm, respectively. Also, an increase in the average strand length from 5 ± 2 μm to 11 ± 4 μm is observed. The differences in nanostrand length can be explained as follows. Tymoczko *et al.*^12^ observed that laser-generated Au_20_Fe_80_ NPs have a higher abundance of CS NPs in contrast to Au-rich solid solution particles than in the case of the Au_50_Fe_50_ system. This also goes along with a much higher mass abundance of magnetic bcc Fe, demonstrated by XRD in that study. Furthermore, the formation of NCS NPs, with an ultrathin Au shell and a large volume fraction of Fe in the core, is exclusively observed in the Au_20_Fe_80_ sample, which indicates its significance in nanostrand formation. In particular, only segregated NPs like CS and NCS, which have a Fe-rich component with a high magnetic moment, can contribute to nanostrand formation. In contrast, solid solution NPs have a lower magnetic moment that is damped by alloying of Fe with Au. These differences in the abundance of highly magnetic NPs may explain the formation of longer strands in case more Fe-rich compositions are used. These findings demonstrate that the strand length changes with the Fe content in the sample and the abundance of CS and NCS NPs (Fig. 4c). Long strands are generally desired from the point of application^9^ as they yield a higher electrical conductivity of the final composite.^10^ Furthermore, the unique ultrastructure with the Au shell protecting the magnetic core against oxidation is highly interesting for the long-term stability of the magnetic composites.

**Fig. 5:**
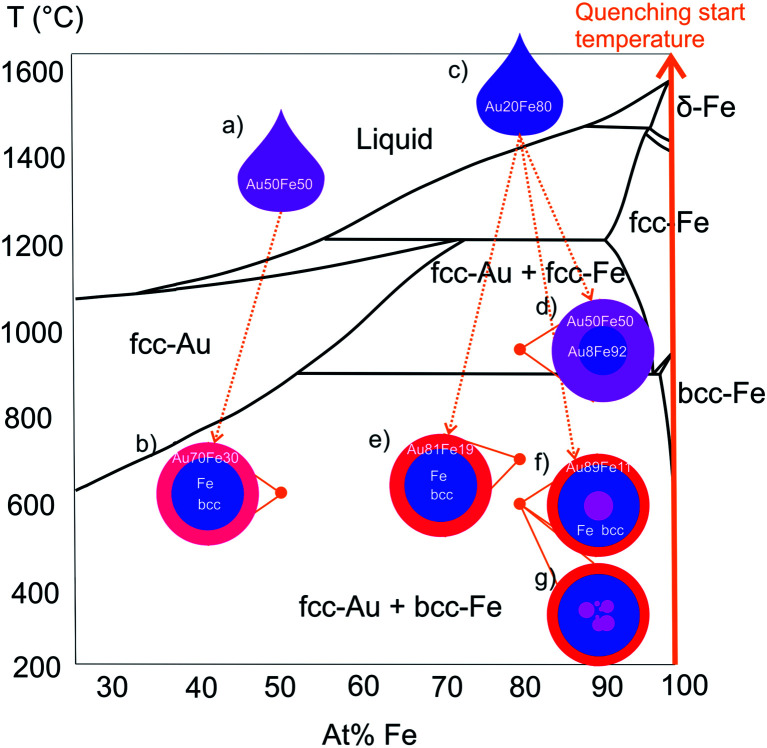
NPs sketches with average chemical composition and crystal structure summarize experimental results. Ultrastructure is assigned to points in the respective section of the bulk phase diagram (redrawn from ref. 33). Formation stats from liquid droplets, depending on their cooling rate different ultrastructure, with the same chemical compositions, are formed (illustrated by arrows). (a) and (c) shows liquid droplets with the same chemical composition as the target and (b), (c)–(g) shows ultrastructures after rapid cooling during particle formation.

The consideration of all described TEM/EDXS results is applied to get an understanding of the formation mechanism of different ultrastructures during LAL of (partly) immiscible alloy targets. According to the experimental results (chemical composition and crystal structure), all characterized NPs may be assigned to a relevant section of the Au–Fe bulk phase diagram (Fig. 5). One primary finding from this work is that all the NPs possess the composition of the bulk target on a single-particle level, which seems to indicate that they all originate from a state where all elements are thoroughly mixed and the bulk target composition determines particle composition. This mechanism was previously hypothesized to occur for LAL with nanosecond pulses,^14^ while deviations were reported to occur in the case of picosecond pulses.^13^ The chemical composition of the individual components is averaged over a total of all NPs with respective ultrastructure, which includes 20 CS NPs (6 NPs for Au_50_Fe_50_, 7 NPs with Au–Fe alloy core, and 7 with pure Fe core for Au_20_Fe_80_) and 3 NCS NPs.

Interestingly, the chemical composition and respective crystal structure of the ultrastructures can be assigned to different metastable high-temperature states in the bulk phase diagram, which was not expected to occur, because LAL is a non-equilibrium synthesis method with very high cooling rates that may generate phases not predicted by the phase diagram.^19^ The bulk phase diagram is considered as a reference for a qualitative comparison of the investigated Au–Fe nanoalloy systems, although the phase diagram for nanoalloys is expected to show derivations due to surface effects.^27^ It should be noted that SoSo NP fractions with mean diameters smaller than 10 nm are formed for Au_50_Fe_50_ and Au_20_Fe_80_ as well, however, they are not the focus of this study, a detailed investigation of their formation conditions is given in Tymoczko *et al.*^12^

Under equilibrium conditions, the segregation, based on immiscibility and drastic difference in surface energies of Au and Fe, would cause the formation of CS NPs with Au-rich shells.^13,28^ However, for both samples, CS NPs with metastable Au–Fe alloy shells are found (Fig. 5b and e). The measured phase composition of core and shell equals the one that would result from bulk quenching experiments, where differences in alloy shell composition could be attributed to specific temperatures at which rapid cooling during particle formation froze metastable phase compositions, as shown in Fig. 5. In this context, it should be noted that the information on quenching temperature is not quantitative as the bulk phase diagram cannot be directly applied for nanostructures. However, these qualitative considerations would allow us to hypothesize that *e.g.* NPs with a thick shell and alloy core (Fig. 5d) are quenched at a higher temperature than other CS structures. Besides these, a complex ultrastructure of NCS NPs with Au-rich ultrathin shell (Fig. 5f and g) is observed, which cannot be explained based on the bulk phase diagram. The absence of NCS NPs and the CS NPs with thick shell (Fig. 5d) for Au_50_Fe_50_ also implies the importance of overall chemical composition in the formation of these ultrastructures. Nevertheless one needs to consider that different laser pulse durations were used for the generation of the Au_50_Fe_50_ (picosecond) and the Au_20_Fe_80_ nanoparticles (nanosecond). Based on previous work^13^ we can assume that the laser pulse duration can influence the core shell yield and the particle diameter, which we were able to verify using thin film targets. In this case, a longer pulse duration in the nanosecond regime, as well as a well-mixed target, were the main effectors yielding a high yield segregated core–shell structures close to thermodynamic equilibrium. Therefore, switching to nanosecond pulsed ablation for the Au_50_Fe_50_ may influence the ultrastructure and CS yield of Au_50_Fe_50_, however, we would instead expect a shift towards thermodynamically-controlled products and hence less complex ultrastructures. Based on this we can conclude that primarily the target composition influences the formation of more complex structures, while we would consider the pulse duration effect less pronounced. This is further backed by our previous experiments where we did not observe any NCS NPs when using 50 : 50 compositions, alloyed targets, and nanosecond pulses during ablation.^12,15^”

The formation mechanism of all ultrastructures is believed to start with liquid droplets with the same total chemical composition as the target (Fig. 5a and c). Theoretical results from atomistic modeling show processes that may be responsible for different thermal conditions during particle formation.^29–31^ These processes refer to phenomena occurring on very short timescales up to a few nanoseconds, and particle formation can be correlated to different thermal quenching rates, resulting from atomistic processes within the formation mechanism:

According to these atomistic calculations from the Zhigilei group conducted for ultrashort pulses,^29–31^ as well as recently for pulse durations up to 2 ns (ref. 32) NPs are created in a metal liquid mixing region that forms at the interface of a transient laser-generated hot molten metal layer and the liquid environment, while three different particle formation mechanisms with significantly deviating quenching rates are proposed. According to Zhigilei *et al.*, the first particle formation mechanism probably occurs at the edge of the expanding ablation plume due to the active evaporation of metal atoms at the hot plume–liquid interface. These atoms are subject to a quick cooling process and quickly crystallize to form small NPs. Due to the high quenching rate and small particle size, this mechanism may cause the formation of solid solution NPs far from thermodynamic equilibrium. Two other mechanisms are predicted to occur in the lower part of the ablation plume, closer to the target. Here the hot molten layer disintegrates during early stages, and penetration (as well as expansion confinement) by the supercritical liquid would lead to the formation of larger droplets that are subject to slow cooling (still in the liquid state after several nanoseconds). A third mechanism occurs below the transient interfacial layer. These particles are in a high-density environment and would be subject to very high temperature which causes the seeds (1–4 nm) to be thermodynamically unstable and therefore evaporate unless their rapid collision and coalescence leads to small (about 5 nm) nanoparticles, while the larger nanoparticles in this region continue to grow. These processes result in thermodynamically stable, mostly larger nanoparticles,^32^ and they would still stay in layers close to the target where cooling rates are relatively low, and particle formation closer to thermodynamic equilibrium conditions are possible. It is conceivable that particles from mechanisms two and three are the origin of the segregated CS structures observed in this study.

Nevertheless, the reason for the formation of the additional Au-rich cores (Fig. 5f and g) in the Fe matrix within the NCS can only be speculated so far and may not be explained solely based on the bulk phase diagram and the stated particle formation mechanism. Differences in size and the chemical composition of the particles are excluded as decisive parameters since CS, and NCS NPs have the same overall chemical composition (Table S1†) and similar average diameters (Fig. S6 and S7†). The existence of a nested core with an Au-rich chemical composition, which is not depicted in the phase diagram, may indicate a formation process accompanied by incomplete agglomeration or diffusion processes. This finding is underlined by the existence of NPs with multiple nested cores (Fig. 5g) identified in HAADF-STEM *Z*-contrast images (Fig. S8†). Although we cannot provide statistically meaningful data on size selectivity between CS and NCS, for the larger particles we only see NCS, and the NCS abundance increases with particle diameter (Fig. S7†), so that larger particle volumes may also support NCS formation.

During NPs formation, particularly when cooling of a liquid metal droplet is considered (mechanism 2), Fe would crystallize first due to its higher melting temperature and would be trapped within liquid Au, which later solidifies to form a shell. In case the main component of the particle is Fe, like in the case of Au_20_Fe_80_, it is possible that small droplets of liquid Au may be kinetically trapped within the crystalizing Fe, in particular at large volumes. If this core matrix is Fe bcc, diffusion of the Au through the core into the shell would be energetically unfavorable, since the surface energy of the Au would have to overcome and hetero-diffusion in Fe bcc is unfavorable. Hence, the nested core may be kinetically stabilized in the Fe matrix. The inclusion of Au in a bcc core could also occur through the transformation of an alloy fcc core (Fig. 5d) to a Fe bcc core.

These findings describe the initial stage of the formation process (first nanoseconds) and therefore do not allow precise conclusions about the full mechanism on an atomistic scale of the formation of complex metastable segregated ultrastructures. Additional effects of cavitation bubble dynamics on particle formation, which usually occur on a time scale from μs to ms, cannot be excluded, for example, during the collapse phase. Furthermore, it should be noted that the atomistic simulations, which are the basis of the stated formation mechanism, were conducted for silver in water, and deviations during transfer to the Au–Fe system in acetone are conceivable.

The generated ultrastructures (Fig. 5) contain phases that are comparable to those that would be formed in bulk quenching experiments according to the Au–Fe phase diagram. However, the results underline the complexity of the formation mechanism of laser-generated bimetallic NPs, in particular at immiscible molar fractions, and the need for further investigations. For this purpose, other material combinations, such as Au–Co or Co–Pt, could be employed. A more detailed investigation of Au–Co NPs could provide significant insights into the formation mechanism of bimetallic NPs due to other material parameters such as a wider two-phase region in the Au–Co phase diagram at high Co molar fraction.^33^

## Conclusion

In conclusion, we applied an advanced electron microscopy characterization technique to a multitude of Au–Fe NPs, which allows the quantification of chemical composition for individual components of complex ultrastructures at the nanoscale. Interestingly, despite the diversity of ultrastructures created, the composition of the laser-generated NPs is always close to the one of the targets used for laser ablation synthesis, which allows the conclusion that the mass-weighted majority of NPs are formed from liquid droplets with negligible deviation from the bulk target composition. We identified single-crystal, zero-valent Fe bcc cores, and crystalline Au–Fe fcc alloy shells (Fe@AuFe) with variable chemical composition and crystallographic Bain orientation relation. In the case of nested core–shell NPs, different Au-content of Au-rich shell and core, respectively, was observed. Under the external magnetic field, these colloidal Fe@AuFe NPs quickly form micrometer-long nanostrands within a polymer matrix, demonstrating potential application of these unique NPs within application areas such as radiation shielding or transparent-conductive coatings.

A formation mechanism, based on different quenching conditions in the early stages of pulsed laser ablation in liquid, is considered to explain unique ultrastructure formation. In dependence on the quenching rate, different phases, in analogy to the bulk phase diagram, originate. We obtained unique experimental findings to develop a model of the formation mechanism of segregated NPs by following recent theoretical findings of molecular dynamic simulations. These insights underline the importance of the phase diagram, which seems to affect the formation mechanism in intermediate steps and may allow the prediction of ultrastructures generated by LAL for other bimetallic systems.

## Conflicts of interest

There are no conflicts to declare.

## Supplementary Material

NA-002-D0NA00514B-s001
