# Supplementary material for: Composition and structure of magnetic high-temperature-phase, stable Fe–Au core–shell nanoparticles with zero-valent bcc Fe core
Source: Nanoscale Adv. 2020 Aug 10;2(9):3912–20. doi: 10.1039/d0na00514b (PMC9417649; doi:10.1039/d0na00514b)
Supplement: NA-002-D0NA00514B-s001 [file NA-002-D0NA00514B-s001.pdf]

Electronic Supplementary Material (ESI) for Nanoscale Advances.
This journal is © The Royal Society of Chemistry 2020

Supporting information

Composition and structure of magnetic high-temperature-phase, stable Fe-Au core-shell nanoparticles with zero-valent bcc Fe core

Marius Kamp[1], Anna Tymoczko[2], Radian Popescu[3], Ulrich Schürmann[1], Ruksan Nadarajah[2], Bilal Gökce[2] Christoph Rehbock[2], Dagmar Gerthsen[3], Stephan Barcikowski[2], and Lorenz Kienle[1*]

1 Institute for Materials Science, Synthesis and Real Structure, Kiel University, Kaiserstraße 2, 24143 Kiel, Germany

2 Technical Chemistry I and Center for Nanointegration Duisburg-Essen (CENIDE), University of Duisburg-Essen, Universitätsstrasse 7, 45141 Essen, Germany

3 Laboratory for Electron Microscopy (LEM), Karlsruhe Institute of Technology (KIT), Engesserstr. 7, 76131, Karlsruhe, Germany

Table S1: Average Fe content from EDXS measurements of single NPs with standard deviation σ, absolute error Δf, and variance $σ^2$.

at% Au	$Au_{20}Fe_{80}$	$Au_{50}Fe_{50}$
average	22.6	50.3
σ	2.4	6.2
Δf	0.5	1.8
$σ^2$	6.0	38.5
Number of NPs	21	12

Table S2: quantified EDXS analyses of single NPs (average compositions) for comparison with the subshell approach. The absolute deviation is ±2.3 at% Au on average, calculated from row 4.

Number of NP	C_1(Subshell approach) Au at%	C_2 (Reference) Au at%	absolute Δc Au at%
1	62	63	0.9
2	52	56	4.0
3	51	53	1.6
4	43	44	1.3
5	52	54	2.0
6	77	78	1.0
7	84	80	4.0
8	83	80	3.0
9	76	78	2.0
10	69	69	0.0
11	71	76	5.0
12	71	74	3.0
13	76	78	2.0

14	79	84	5.0
15	72	75	3.0
16	69	72	3.0
17	83	81	2.0
18	74	75	1.0
19	75	74	1.0
20	71	73	2.0
21	79	77	2.0
22	77	76	1.0
23	86	85	1.0
24	72	76	4.0

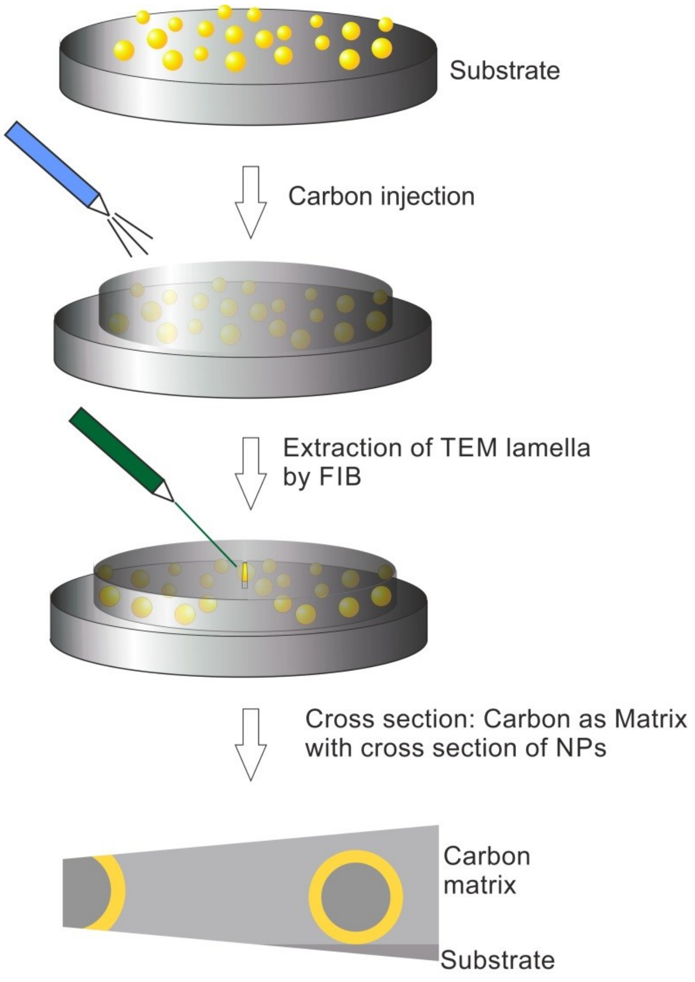

Fig. S1: Sketch of the steps of FIB cross-section preparation of NPs. NPs are embedded in C-Matrix before standard lift-out preparation. At the tip of the lamella, a section is extracted from a core-shell (CS) NP.

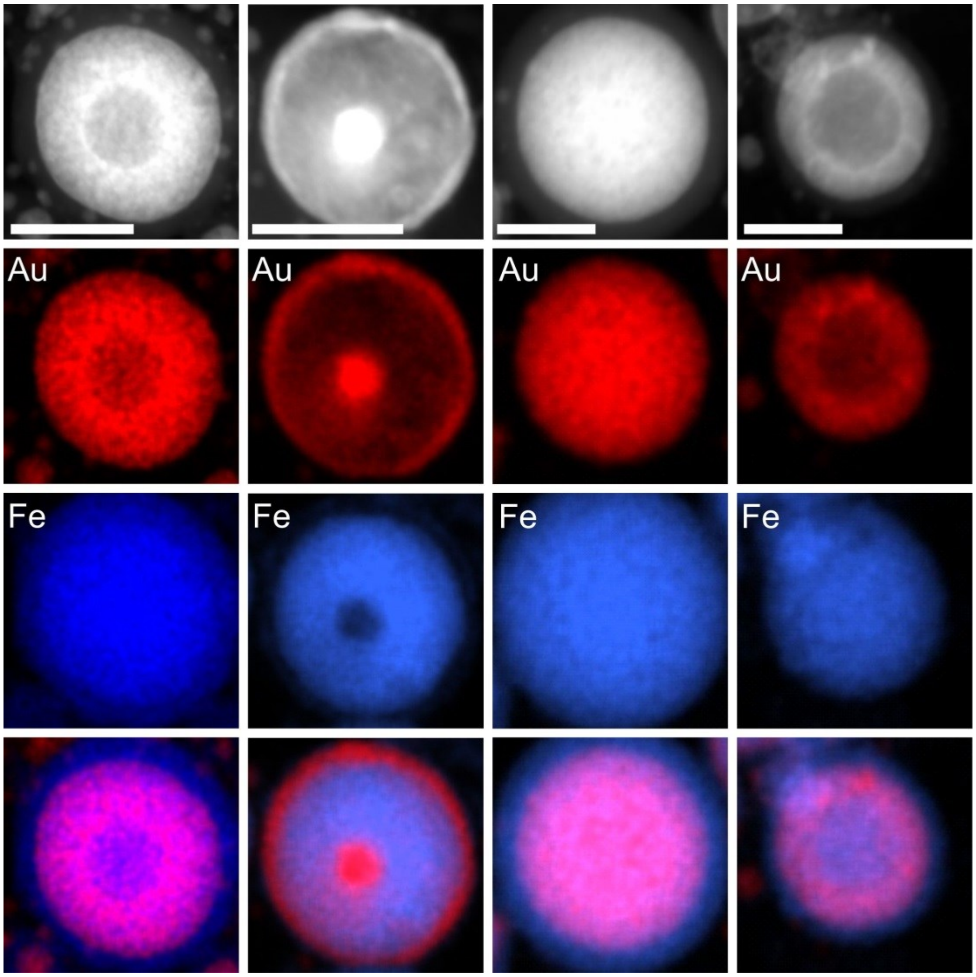

Fig. S2: (Top row) HAADF-STEM Z-contrast images and (bottom rows) elemental maps for Au (Au-$L_{\alpha1}$ line) and Fe (Fe-$K_{\alpha1}$ line). The overlay of the Fe and Au signals illustrates the of an outermost Fe-containing (presence of a FeO_x) deposit on the CS and solid solution NPs, probably stemming from FeO_x by-products present in the liquid before drying.

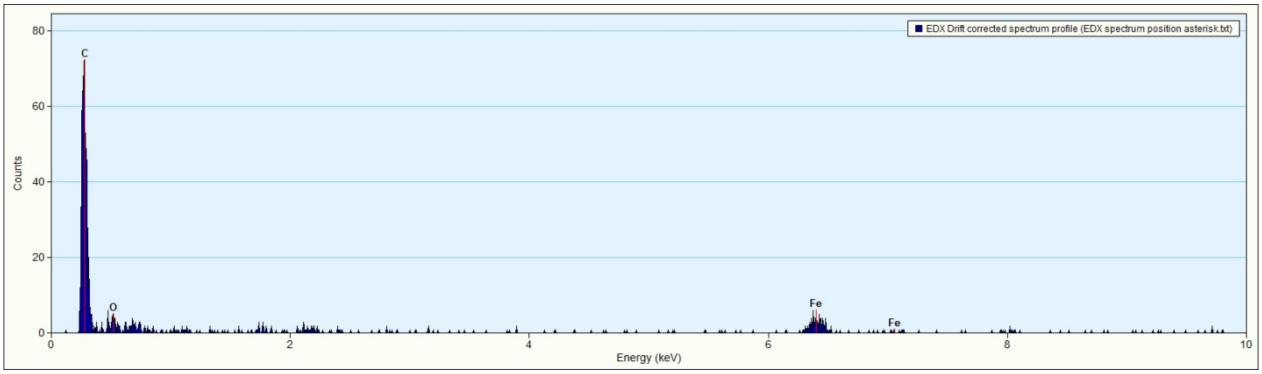

Fig. S3: EDXS spectrum of the position marked by an asterisk in Fig. 4. Chemical composition is quantified to be 100 at% Fe. Carbon and oxygen signal arises from the carbon matrix, that is needed to stabilize the sample during sample preparation.

$$d_f = d_i + 0.05 \cdot \frac{Z_m}{E_0} \cdot \left(\frac{\rho}{A_m}\right)^{\frac{1}{2}} \cdot D^{\frac{3}{2}} * \left(\frac{\rho}{A_m}\right)^{\frac{1}{2}} * D^{\frac{3}{2}} \qquad (1)$$

Electron beam broadening was calculated by the Goldstein approach using Eq. (1), which yields the broadened beam diameter d_f for an electron beam with an initial probe diameter d_i and energy E_0 in *keV* after passing through a NP characterized by an average atomic number Z_m, an average atomic mass A_m, an average density ρ_m in $\frac{g}{cm^3}$, and a sample thickness (NP diameter) D in *nm* [1]. For our transmission electron microscope without aberration corrector, a probe diameter of d_i=0.4 nm is reasonable. For electron energy of 200 keV, Eq. (1) yields a broadened beam with d_f=0.80 nm for NPs with maximum diameter D=29 nm and nominal composition of $Au_{20}Fe_{80.}$ Beam broadening up to 0.78 nm is expected for NPs with D=23 nm, and the average composition of $Au_{50}Fe_{50}$. Accordingly, the distance between two adjacent measuring points along EDXS line scans was chosen to be of 1 nm (i.e. larger than d_f-values) to avoid overlap of the EDXS signal recorded from two neighbor regions of the NP.

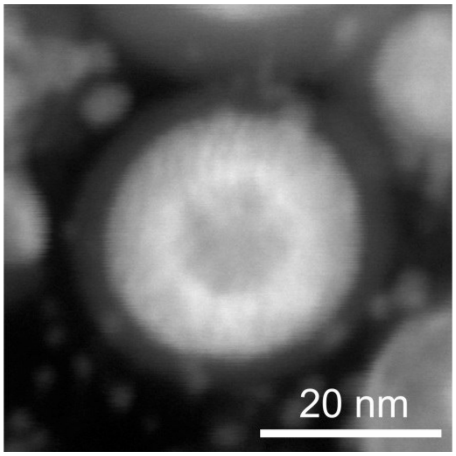

Fig. S4: HAADF-STEM Z-contrast image of the CS NP depicted in Fig. 3 d)-f).

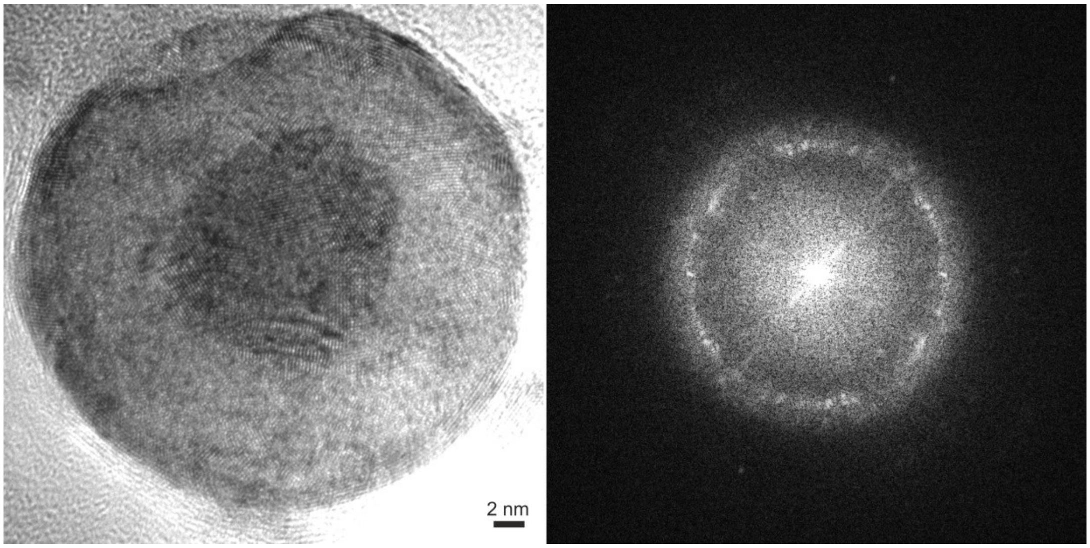

Fig. S5: HRTEM (left) and Fast Fourier Transform (right) of NCS NPs, showing the polycrystalline shell.

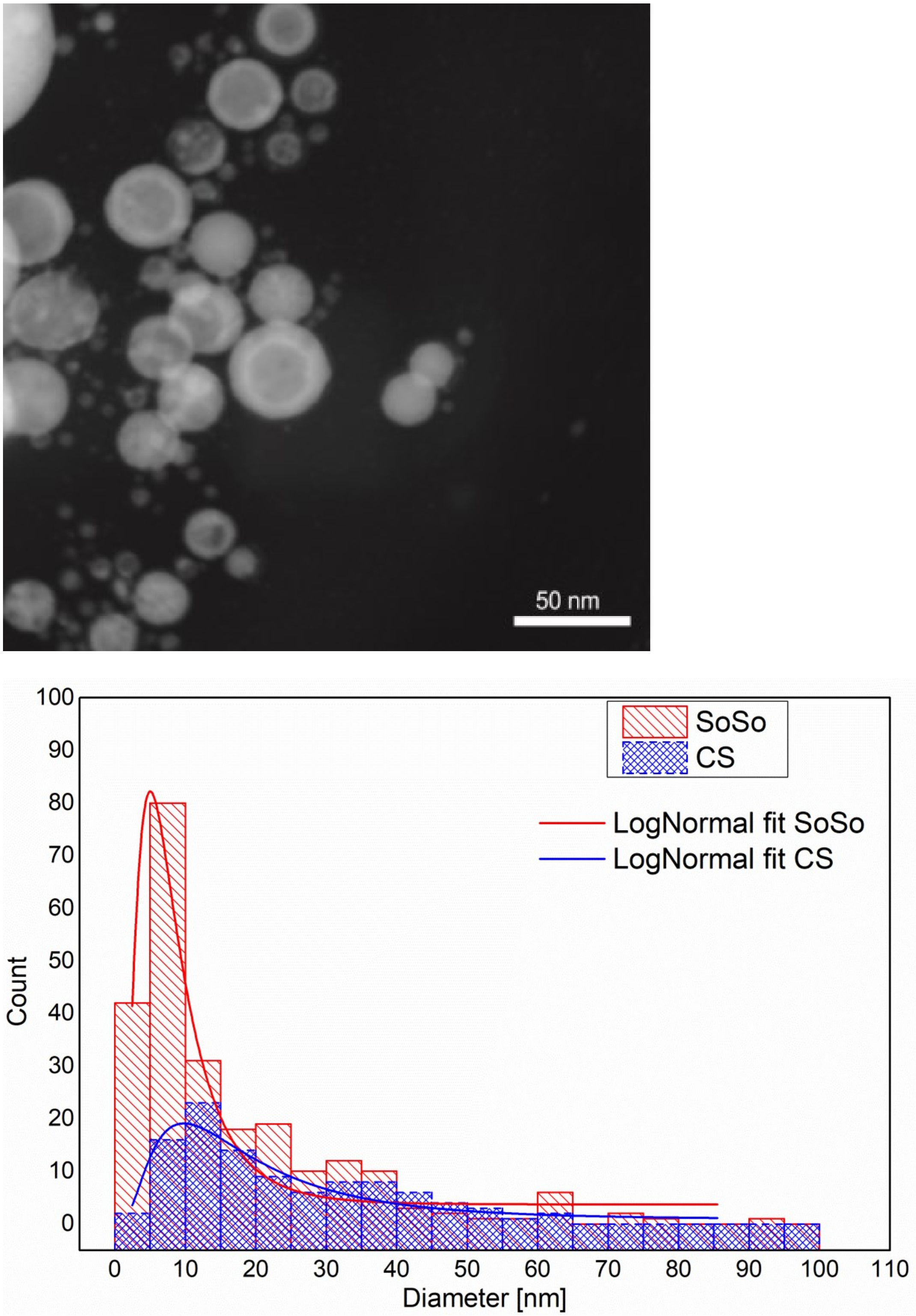

Fig. S6: Overview HAADF-STEM Z-contrast image and representative size distribution for $Au_{50}Fe_{50}$ solid solution and CS NPs and respective LogNormal fit. Resulting average size (xc) SoSo = 8 nm, CS = 19 nm.

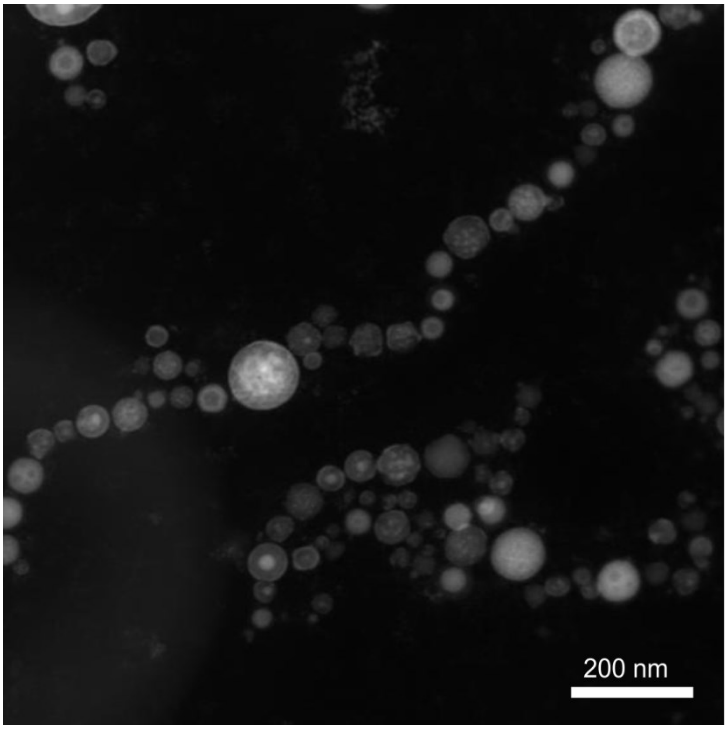

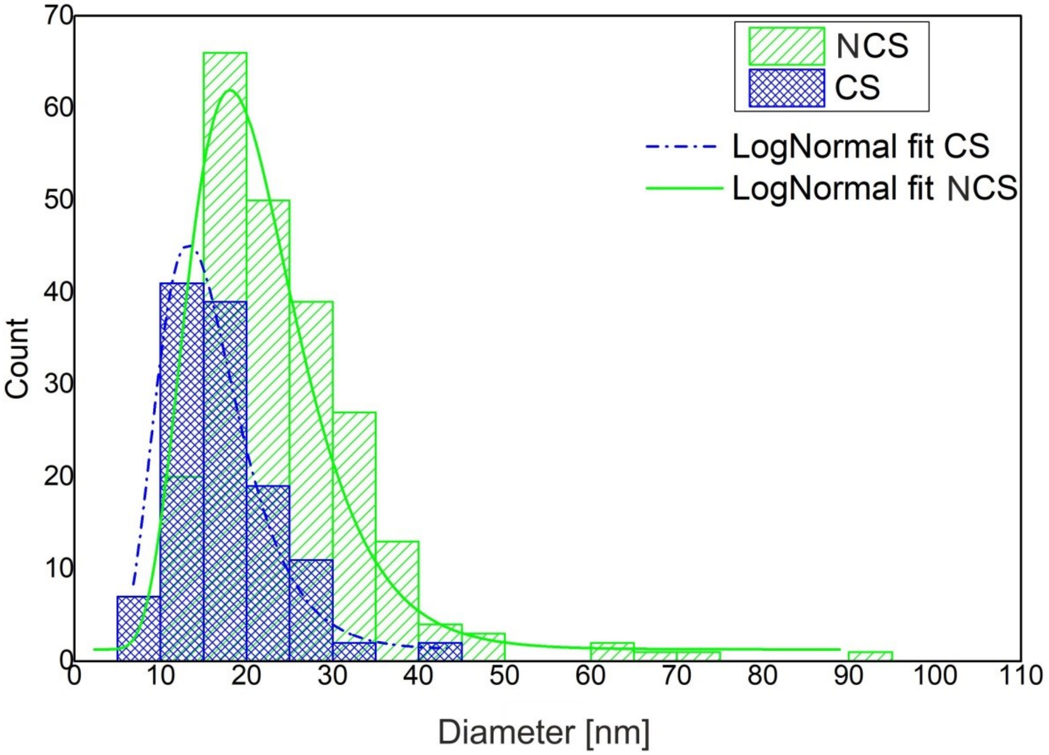

Fig. S7: Overview HAADF-STEM Z-contrast image and representative size distribution of NCS and CS NPs from ($Au_{20}Fe_{80}$) and respective LogNormal fit. Resulting average size (xc) NCS = 16 nm, CS = 22 nm. Also, the sample contains SoSo NPs that are not shown in this graph.

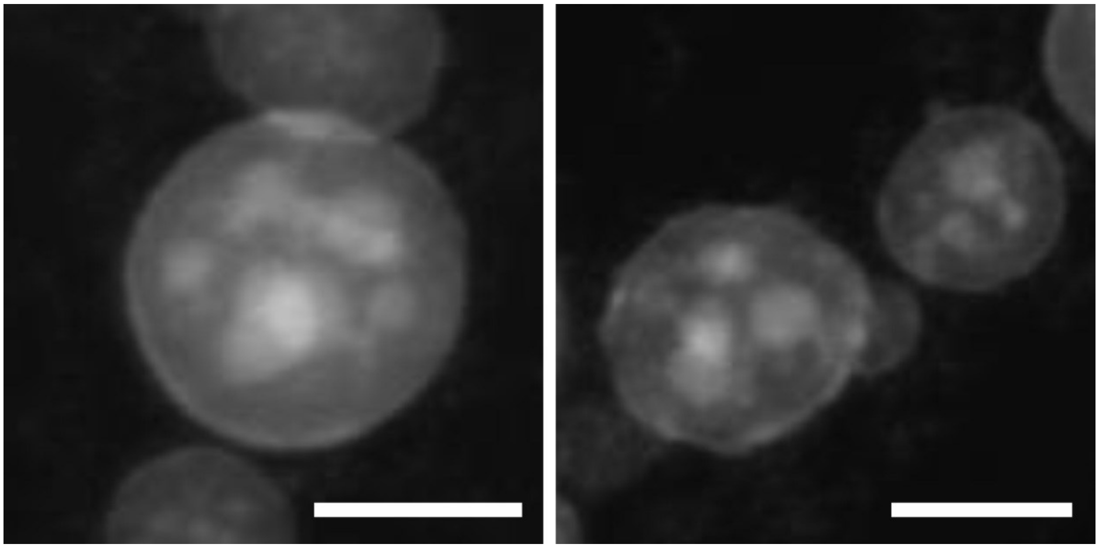

Fig. S 8: HAADF-STEM Z-contrast image of NCS NPs with fragmented nested cores. Scale bars are 25 nm.

References

1 S. Barcikowski, T. Baranowski, Y. Durmus, U. Wiedwald, and B. Gökce, *J. Mater. Chem. C*, 2015, **3**, 10699–10704.

2 J. I. Goldstein, D. E. Newbury, J. R. Michael, N. W. M. Ritchie, J. H. J. Scott, and D. C. Joy, *Scanning Electron Microscopy and X-Ray Microanalysis*, Springer-Verlag, New York, 4th, 2018.
